# Supplementary material for: Comparative analysis of Wolbachia maternal transmission and localization in host ovaries
Source: Commun Biol. 2024 Jun 14;7:727. doi: 10.1038/s42003-024-06431-y (PMC11178894; doi:10.1038/s42003-024-06431-y)
Supplement: Supplementary file 2 — Description of Additional Supplementary Files [file 42003_2024_6431_MOESM2_ESM.pdf]

Description of Additional Supplementary Files

File name- Supplementary Data 1

File description- Source data behind the analysis of maternal transmission.

File name- Supplementary Data 2

File description- - Source data (Ct values) behind the analysis of Wolbachia densities in host tissues.

File name- Supplementary Data 3

File description- Source data (CTCF values) behind the analysis of Wolbachia abundance in stage 10 oocytes.
